# Pointless pondering on predatory publications 

**DOI:** 10.6026/97320630016342

**Published:** 2020-04-30

**Authors:** Pandjassarame Kangueane, Senkuttuvan Pavithra

**Affiliations:** 1 Biomedical Informatics Private Limited, Irulan Sandy Annex, Pondicherry 607 402

**Keywords:** Predatory, greedy, publications, journals, ISSN, DNS, caveat Emptor

## Abstract

It is a pointless pondering (thinking) on predatory (meaning greedy) publications (meaning journals) while practicing publishing through freedom of expression and or the Press where applicable. It should be noted that a weak publication will vanish (disappear) itself in an open access publishing model where contents are made available for free on the WWW. The fundamental question in this context is the definition of host (congregation) and predator (intruder). The second question is the type (data and or commercial) and subsequent measure of effect of the predator on the host. Detailed discussion on this issue or any other related issue is welcomed under the freedom of the Press yet conclusion on it will be often biased and is clearly unwarranted. The parties aware of such concerns should write to the publisher (with address for communication) to take such action within such time to stand corrected. Please be informed that ISSN is unique for each publication and portals for ISSN is distributed throughout the world in each country. This is well monitored and clearly streamlined. Therefore, NO two publication titles will be identical. Awareness from authors on misleading or misinformed or misrepresented ISSN is important and such information should be petitioned to ISSN and portals for ISSN that is distributed throughout the world with state mechanisms to monitor such activities. Academia should be self-aware on these issues and have discussions on the quality and quantity of data taken to the context. Caveat Emptor is applicable to a considerable extend among the literate community as in this case. The only problem could arise because of compromised (unregistered or mirrored) ISSN number published on the WWW which is already well regulated through DNS lookup. Therefore, parties concerned about ethical issues on scientific publishing should write to concerned publishers with known address to stand corrected or to ISSN and portals for ISSN or to DNS lookup where address is not available to correct such issues through available state mechanisms. Hence, biased advisory notes from government representations, society sponsored mass campaign through news/TV media and academic miss representation based on data collected by an individual without physical address for communication is clearly unwarranted in this regard.

## Description

Science for service in a society is the success story of scientists with serenity [1]. Hence, scholarly communication is critical. Open access to known literature is critical in developing technologies for serving the society [2].

*“Availability, accessibility and applicability of known scientific literature data is essential”.*

Publishers provide platforms to communicate scholarly materials by the formation, development and maintenance of journals with the help of adequate editorial and (or) peer-reviews where possible. This is a mammoth task and it is often non-trivial. Resources (software and hardware), investment and man hours are involved in this endeavour. Thus, development of a platform (media) for scholarly communications is noble. 

*“An idea becomes an asset in this process”*

The famous data analytics company Clarivate Analytics Inc. [3] uses ~ 28 stringent parameters for the inclusion of a journal in their database. Therefore, there are non-linear models and mechanisms to streamline scholarly publications that are made available over the internet across the globe. 

It should be noted that ISSN [4] is unique for each publication and portals for ISSN is distributed throughout the world in each country. This is well monitored and clearly streamlined. Therefore, NO two publication titles will be identical. Awareness from authors on misleading or misinformed or misrepresented ISSN is important and such information should be petitioned to ISSN and portals for ISSN that is distributed throughout the world with state mechanisms to monitor such activities. Academia should be self-aware on these issues and have discussions on the quality and quantity of data taken to the context. Caveat Emptor is applicable to a considerable extend among the literate community as in this case. 

*“Engineering and technological developments using science data for service in the society are vital. This is possible through access to science data”*

Compromised (unregistered or mirrored) ISSN number published on the WWW is already well regulated through DNS lookup [5]. The parties aware of such concerns should write to the publisher (with address for communication) to take such action within such time to stand corrected. The fundamental question in this context is the definition of host (congregation) and predator (intruder). It should be noted that there are state mechanisms to monitor such issues. Detailed discussion on this issue or any other related issue is welcomed under the freedom of the expression (and (or) Press) yet conclusion on it will be often biased and is clearly unwarranted.

*“Debate without communication is impossible”*

Parties concerned about ethical issues [6] on scientific publishing should write to concerned publishers with known address to stand corrected or to ISSN and portals for ISSN or to DNS lookup where address is not available to correct such issues through available State mechanisms. Hence, biased advisory notes from government representations, society sponsored mass campaign through news/TV media and academic miss representation based on data collected by an individual without physical address for communication is clearly unwarranted in this regard. Thus, a weak publication will vanish (disappear) itself in an open access publishing model where contents are made available for free on the World Wide Web (WWW). Hence, it is a pointless pondering (thinking) on predatory (meaning greedy) publications (meaning journals) while practicing publishing through freedom of expression (and or the Press) where applicable.

*“Timely communication of scholarly materials is important”*
